# Electrocardiographic changes in QTc interval and other parameters associated with osimertinib therapy

**DOI:** 10.3389/fonc.2025.1612758

**Published:** 2025-06-18

**Authors:** Yilin Pan, Kexin Peng, Yichuan Jiang, Ping Yang, Beibei Du, Yuquan He

**Affiliations:** ^1^ Department of Cardiology, China-Japan Union Hospital of Jilin University, Jilin Provincial Cardiovascular Research Institute, Changchun, China; ^2^ Department of Critical Care Medicine, Beijing Anzhen Hospital, Capital Medical University, Beijing, China; ^3^ Department of Pharmacy, China-Japan Union Hospital of Jilin University, Jilin Provincial Cardiovascular Research Institute, Changchun, China

**Keywords:** osimertinib, QTc interval, electrocardiographic changes, cardiotoxicity, cardio-oncology

## Abstract

**Introduction:**

Osimertinib, a third-generation EGFR-TKI, is approved for the first-line treatment of EGFR-mutated advanced NSCLC and for T790M-positive NSCLC after progression on previous EGFR-TKI therapy. Osimertinib is associated with cardiotoxicity, including severe QT interval prolongation, which necessitates detailed evaluation due to its increasing use.

**Methods:**

This retrospective, single-center study conducted at Jilin University China-Japan Union Hospital evaluated electrocardiographic changes with EGFR-mutated NSCLC treated with osimertinib. The study consecutively included 102 patients diagnosed with NSCLC at the China-Japan Union Hospital of Jilin University from August 1, 2019, to December 31, 2022. Among them, 41 patients who had both pre- and post-treatment ECG records while receiving osimertinib (80 mg daily) were analyzed. ECG parameters such as heart rate, PR interval, QRS duration, QRS axis, and QT intervals were manually calculated and analyzed.

**Results:**

The study population had a mean age of 68.56 ± 8.73 years, comprising 46.34% males. Significant changes were observed in the QTc Bazett interval (*P* < 0.0001), QTc Fridericia interval (*P* = 0.0002) and QRS axis (*P* = 0.0387) post-treatment. QT interval prolongation was significant (McNemar test *P* = 0.0039), with both QTc Bazett and QTc Fridericia intervals showing notable prolongation. However, the changes in heart rate, PR interval, and QRS duration were not statistically significant.

**Conclusion:**

This study comprehensively investigates the electrocardiographic changes before and after osimertinib treatment in patients with EGFR-mutated NSCLC. The significant prolongation of QTc intervals underscores the need for rigorous ECG monitoring to mitigate potential cardiotoxicity.

## 1 Introduction

Osimertinib is a third-generation epidermal growth factor receptor (EGFR) tyrosine kinase inhibitor (TKI) (1). Osimertinib has been approved globally for the first-line treatment of patients with EGFR-mutated (EGFRm) advanced non-small cell lung cancer (NSCLC) and for those with NSCLC who have progressed after EGFR-TKI treatment and are T790M-positive. In the FLAURA and AURA studies, osimertinib exhibited manageable and consistent tolerability characteristics (2–5).

Similar to many antitumor drugs, osimertinib also causes several adverse reactions. Common adverse events include diarrhea, rash, dry skin, and paronychia (2). Cardiotoxicity, though less common, is a significant adverse effect (6–8). With the increasing use of osimertinib in postoperative adjuvant chemotherapy and combination therapy, its cardiotoxicity has become a significant concern (9, 10). In a previously published case report by our team, a patient treated with experienced severe QT interval prolongation,ventricular arrhythmias, and syncope, indicating its potential to cause serious cardiac adverse reactions (11).

Compared to gefitinib and erlotinib, osimertinib appears to have a higher incidence of cardiac-related adverse events. Anand K et al. (12) found through analysis of the FDA Adverse Event Reporting System that among the 315 cardiac adverse events related to EGFR-TKIs, osimertinib accounted for more than half (158 cases, 50.1%). The most common cardiac adverse event was heart failure, followed by QT interval prolongation. However, the Adverse Event Reporting System has limitations, such as the lack of baseline cardiac characteristics of patients. Ewer et al. (13), based on analyses of three large databases including FLAURA and AURA3, concluded that there is no correlation between osimertinib and the development of heart failure. However, the relationship between osimertinib and QT interval prolongation remains unclear, as no prior studies have systematically evaluated ECG changes before and after treatment in patients. These findings prompted a detailed single-center retrospective analysis to better understand osimertinib-related cardiac adverse events in EGFR-mutant NSCLC patients.

## 2 Method

### 2.1 Patients selection

This retrospective, single-center, observational study was conducted at the China-Japan Union Hospital of Jilin University, approved by the Ethics Committee of China-Japan Union Hospital of Jilin University (Approval No: 20220118014), and conducted in accordance with the Declaration of Helsinki. Throughout the study, patient data were kept strictly confidential. The study included patients diagnosed with NSCLC at the China-Japan Union Hospital of Jilin University from August 1, 2019, to December 31, 2022, whose gene testing showed EGFR T790M mutation positivity and who regularly used osimertinib mesylate (80 mg daily) for at least one cycle. Patients without electrocardiogram records before and after medication, those unwilling to follow up, or those who did not agree to participate in the study were excluded, resulting in a total of 41 patients being included.

### 2.2 Data acquisition

First, detailed inquiries were made through telephone follow-ups regarding the patient’s medical history, smoking history, long-term medication history, and the duration of osimertinib use. Second, the basic information of enrolled patients, such as gender, age, and BMI, was recorded. Electrocardiograms from the electronic medical records before osimertinib use, during use, or within 28 days after discontinuation were extracted, recording heart rate, heart rhythm, PR interval, QRS duration, QRS axis, QT interval, and QT interval corrected by Bazett and Fridericia. The Bazett and Fridericia formulas use the square root and cube root of heart rate, respectively, to correct the impact of heart rate on the QT interval, more accurately reflecting the drug’s effect under constant heart rate conditions. These electrocardiogram parameters were then manually calculated by a doctor with more than 10 years of experience in cardiology. If there was a greater than 20% difference between automatic and manual measurements, the result was adjudicated by a doctor with more than 15 years of experience in cardiology. Osimertinib-related adverse events were graded according to the Common Terminology Criteria for Adverse Events (CTCAE) version 5.0.

### 2.3 Statistical analysis

All data were analyzed using IBM SPSS Statistics version 25.0 software. Measurement data were tested for normal distribution using the Kolmogorov-Smirnov test. Measurement data conforming to a normal distribution are expressed as mean ± standard deviation ( $\bar{\text{x}}$ ± s), and comparisons of parameters before and after medication were performed using paired t-tests. Measurement data not conforming to a normal distribution are expressed as median [M (P_25_, P_75_)], and comparisons between two groups were performed using the Wilcoxon signed-rank test. Count data were expressed as rates and composition ratios and analyzed using the chi-square test. For paired binary data, the McNemar test was used to analyze the significance of differences in prolongation before and after medication. *P* < 0.05 was considered statistically significant.

## 3 Result

### 3.1 Patient characteristics

Of the 102 patients included in the study, 41 had both pre- and post-treatment ECG records. The average age of these 41 patients was 68.56 ± 8.73 years, with 46.34% being male, and an average body mass index of 23.47 ± 3.38 kg/m^2^.Regarding medical history, 31.70% were smokers, 14.63% had hypertension, 2.44% had diabetes, 36.59% had hyperlipidemia, and 9.76% had coronary artery disease. In terms of baseline medication, 2.44% of patients used ACEI/ARB/ARNI, 4.88% used β-blockers, 4.88% used calcium channel blockers, 7.32% used antiplatelet drugs, 2.44% used anticoagulants, 7.32% used statins, and 2.44% used antiarrhythmic drugs. No other QT interval-prolonging drugs were used. The baseline characteristics of these patients are shown in **Table 1**.

**Table 1:** Baseline patient demographics.

Baseline Patient Demographics	Value
Male sex	19 (46.34%)
Age, mean years	68.56 ± 8.73
Body mass index, kg/m^2^	23.47 ± 3.38
Ever smokers	13 (31.70%)
Past Cardiac History	
Hypertension Diabetes Mellitus Hyperlipidemia Coronary Artery Disease	6 (14.63%) 1 (2.44%) 15 (36.59%) 4 (9.76%)
Baseline medications	
Angiotensin converting enzyme inhibitors/angiotensin II receptor blockers/angiotensin receptor-neprilysin inhibitors	1 (2.44%)
Beta blockers	2 (4.88%)
Calcium channel blockers	2 (4.88%)
Antiplatelet Drugs Anticoagulants Statins Antiarrhythmics	3 (7.32%) 1 (2.44%) 3 (7.32%) 1 (2.44%)
Other QT-prolonging drugs	0 (0%)

Data are presented as n (%) for categorical variables and mean ± standard deviation for continuous variables. Body mass index was calculated as weight in kilograms divided by height in meters squared.

### 3.2 Changes in ECG parameters with osimertinib

**Figure 1:**
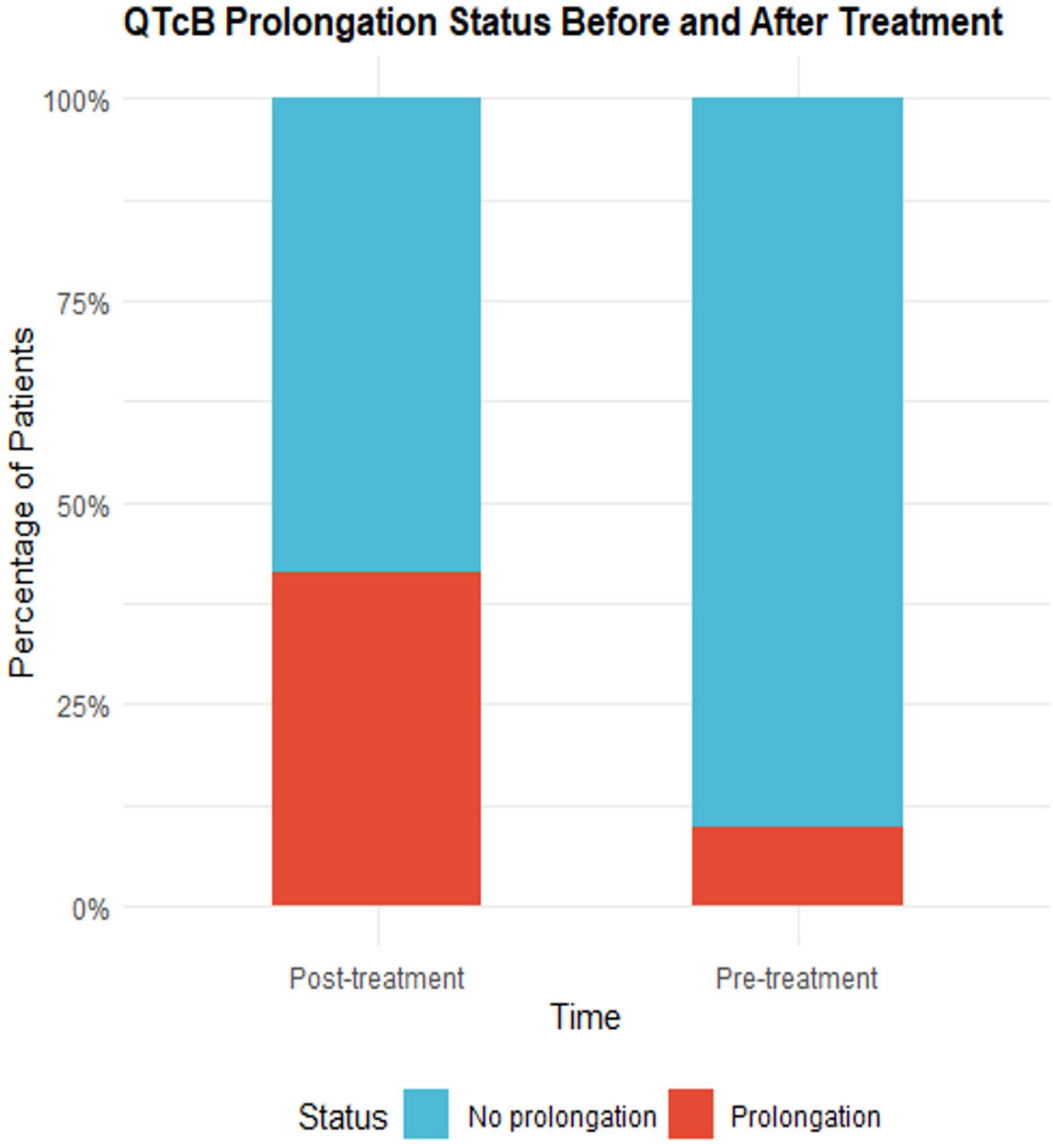
Box plots of electrocardiography parameter changes before and after osimertinib treatment.

In this study, we compared the changes in electrocardiogram parameters before and after osimertinib use. The baseline data included 41 patients; however, due to one patient having a sinus rhythm before medication and atrial fibrillation after medication, the PR interval analysis for this patient could not be performed. Consequently, the PR interval analysis included only 40 patients. The analysis of other electrocardiogram parameters still included 41 patients. The results showed no statistically significant changes in the following parameters: heart rate (*P* = 0.1466), PR interval (*P* = 0.1368), QRS duration (*P* = 0.4713), and QT interval (*P* = 0.3945). However, some parameter changes were statistically significant, including QRS axis (*P* = 0.0387), QTc Bazett interval (*P* < 0.0001), and QTc Fridericia interval (*P* = 0.0002). The median prolongation for QTc Bazett was 18.64 ms, and for QTc Fridericia, it was 12.59 ms. Detailed results are shown in **Table 2**, **Figure 1**.

**Table 2:** Changes in electrocardiography parameters associated With osimertinib exposure.

Electrocardiography parameters	Pre-treatment	Post-treatment	*P* value
Heart rate(per minute)	75.00 (67.00, 91.00)	80.00 (72.00, 96.00)	0.1466
PR interval(ms)	157.43 ± 17.33	152.18 ± 21.62	0.1368
QRS duration(ms)	90.00 (84.00, 98.00)	94.00 (80.00, 102.00)	0.4713
QRS axis(degree)	46.00 (6.00, 60.00)	20.00 (-3.00, 45.00)	**0.0387**
QT interval(ms)	373.61 ± 32.90	378.27 ± 28.70	0.3945
QTc Bazett(ms)	422.49 ± 21.29	442.74 ± 29.32	**<0.0001**
QTc Fridericia(ms)	405.05 ± 17.62	419.68 ± 22.13	**0.0002**

Data are presented as mean ± standard deviation for normally distributed variables and median (interquartile range) for non-normally distributed variables. QTc Bazett, QT interval corrected using Bazett’s formula; QTc Fridericia, QT interval corrected using Fridericia’s formula. Bold P values indicate statistical significance (*P* < 0.05).

**Figure 2:**
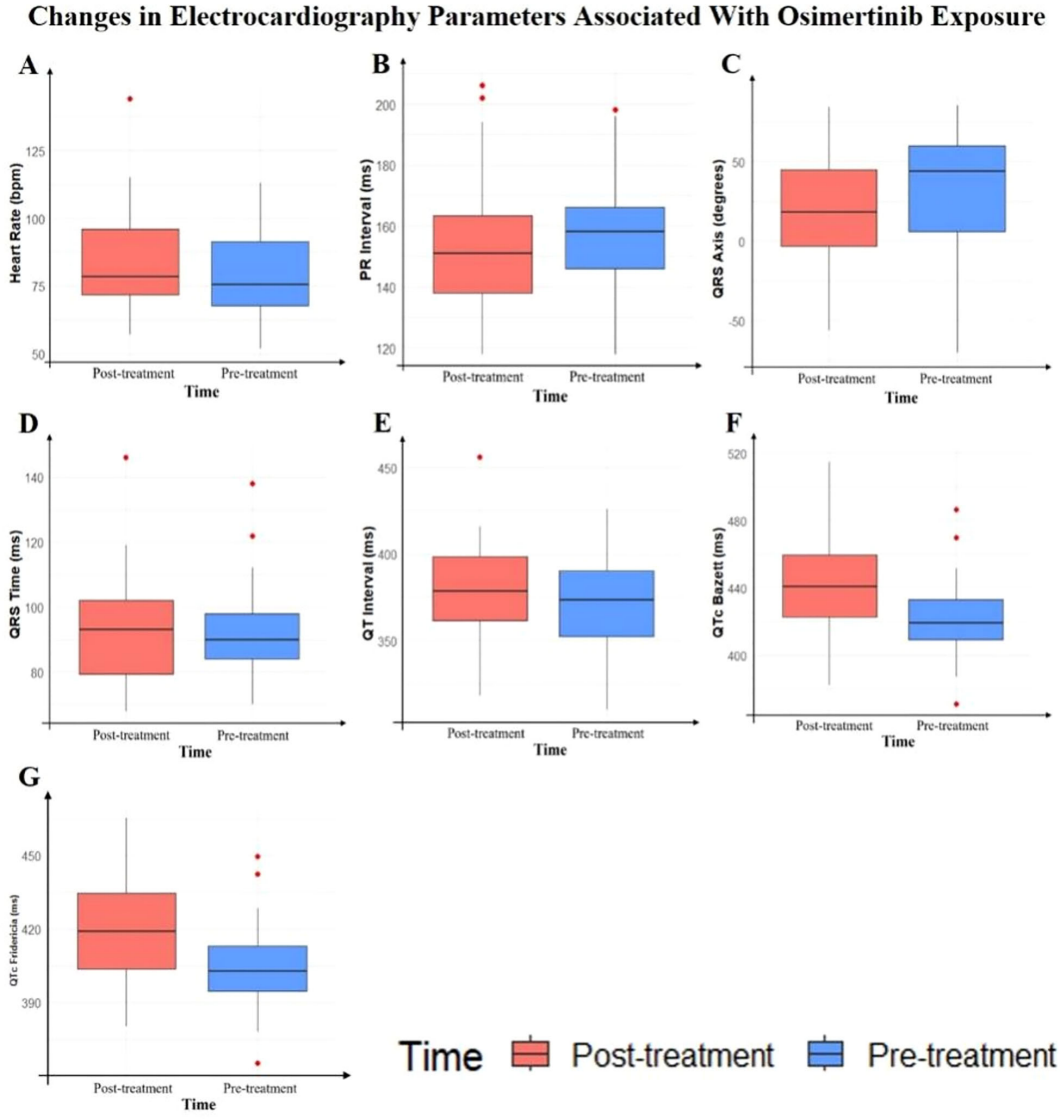
Box plots showing changes in electrocardiography parameters before and after osimertinib treatment. **(A)** Heart rate, **(B)** PR interval, **(C)** QRS axis, **(D)** QRS duration, **(E)** QT interval, **(F)** QTc Bazett, **(G)** QTc Fridericia. Red boxes represent post-treatment values and blue boxes represent pre-treatment values. Red dots indicate outliers.

According to the CTCAE 5.0 standard, the QT interval corrected using Bazett’s formula(QTcB) intervals before and after medication were classified as prolonged or non-prolonged and analyzed using the McNemar test. In three patients, the QTcB prolongation reached grade 3 after medication (an increase of 64 ms, 68 ms, and 108 ms compared to baseline QTcB, respectively). Additionally, there were 37 patients (90.24%) without QTcB prolongation before medication, which decreased to 24 patients (58.54%) after medication. Correspondingly, there were 4 patients (9.76%) with QTcB prolongation before medication, which increased to 17 patients (41.46%) after medication. The P-value of the McNemar test was 0.0039, indicating that the change in QTcB prolongation before and after medication was statistically significant. Detailed results are shown in **Table 3** and **Figure 2**.

**Table 3:** QTcB Prolongation status before and after treatment.

Category	Pre-treatment	Post-treatment	*P* value
No prolongation	37 (90.24%)	24 (58.54%)	**0.0039**
Prolongation	4 (9.76%)	17 (41.46%)	

Data are presented as n (%). QTcB = QT interval corrected using Bazett’s formula. Prolongation was defined according to Common Terminology Criteria for Adverse Events (CTCAE) version 5.0. P value was calculated using McNemar test for paired nominal data. Bold P value indicates statistical significance (*P* < 0.05).

## 4 Discussion

This study comprehensively investigates the electrocardiographic changes before and after osimertinib treatment in patients with EGFR-mutated NSCLC. Our study shows that the corrected QTc Bazett and QTc Fridericia intervals exhibited statistically significant changes.

In the AURA3 study, 1.8% of patients (n=5) had an increase in QTc of more than 60 ms from baseline, and 1.4% of patients (n=4) had QTc exceeding 500 ms after medication; whereas, in our study, these rates were 7.3% and 2.4%, respectively, indicating higher incidence rates (2). Additionally, although the AURA3 study and our retrospective study did not find malignant arrhythmias related to QT interval prolongation, previous case reports have repeatedly recorded instances of ventricular tachycardia and even torsades de pointes in NSCLC patients treated with osimertinib (11, 14–17). These findings suggest that the risk of fatal arrhythmias induced by osimertinib may be underestimated. Physicians should be vigilant about the possibility of such rare malignant arrhythmias in patients using osimertinib and conduct electrocardiogram monitoring for these patients.

The mechanism of QTc prolongation caused by osimertinib is not yet fully understood. Pharmacological and toxicological studies have confirmed that osimertinib affects the cardiac repolarization process by inhibiting the cardiac potassium ion channel Kv11.1 (18). Additionally, animal model studies have shown that downregulating the PI3K signaling pathway through tyrosine kinase inhibition can affect multiple ion channels, including delayed rectifier potassium currents, L-type calcium currents, and sodium currents. This may also be one of the mechanisms by which osimertinib causes QTc prolongation (19).

This study has several limitations that need to be considered. First, this is a small-scale retrospective study conducted at a single center with a small sample size. This patient population does not routinely obtain baseline electrocardiograms, and there is currently no standardized protocol for ECG monitoring and follow-up, which may lead to selection bias. Second, the QT interval is greatly influenced by activity and circadian rhythm changes, which may cause confounding bias. However, all enrolled electrocardiograms were obtained during waking hours, and standardized heart rate correction formulas were used, which should minimize these potential confounding biases. Additionally, as a retrospective study, we were unable to directly correlate ECG changes with the patients’ clinical symptoms at the time, which is a limitation. Future prospective studies are needed to further evaluate the clinical implications of QT prolongation.

QTc prolongation, particularly when exceeding 500 ms, requires careful clinical management. The 2022 ESC guidelines recommend close monitoring for patients with QTc >500 ms, and suggest the use of magnesium agents and beta-blockers to manage the risk of torsades de pointes; for cancer therapy-induced QTc prolongation, normalizing electrolytes and minimizing QT-prolonging drug use are critical strategies; these guidelines also recommend considering beta-blockers if cancer therapy is associated with cardiac toxicity (20).

This study comprehensively examines electrocardiographic changes before and after osimertinib treatment in patients with EGFR-mutated NSCLC. Our findings demonstrate that osimertinib significantly prolongs QTc intervals in these patients. These results emphasize the importance of ECG monitoring to prevent arrhythmic events in patients receiving osimertinib therapy.

## Data Availability

The raw data supporting the conclusions of this article will be made available by the authors, without undue reservation.

## References

[1] Ramalingam SS Vansteenkiste J Planchard D Cho BC Gray JE Ohe Y. Overall survival with osimertinib in untreated, EGFR-mutated advanced NSCLC. N Engl J Med. (2020) 382:41–50. doi: 10.1056/NEJMoa1913662 31751012

[2] Mok TS Wu YL Ahn MJ Garassino MC Kim HR Ramalingam SS. Osimertinib or platinum-pemetrexed in EGFR T790M-positive lung cancer. N Engl J Med. (2017) 376:629–40. doi: 10.1056/NEJMoa1612674 PMC6762027 27959700

[3] Soria JC Ohe Y Vansteenkiste J Reungwetwattana T Chewaskulyong B Lee KH. Osimertinib in untreated EGFR-mutated advanced non-small-cell lung cancer. N Engl J Med. (2018) 378:113–25. doi: 10.1056/NEJMoa1713137 29151359

[4] Goss G Tsai CM Shepherd FA Bazhenova L Lee JS Chang GC. Osimertinib for pretreated EGFR Thr790Met-positive advanced non-small-cell lung cancer (AURA2): a multicentre, open-label, single-arm, phase 2 study. Lancet Oncol. (2016) 17:1643–52. doi: 10.1016/s1470-2045(16)30508-3 27751847

[5] Jänne PA Yang JC Kim DW Bazhenova L Lee JS Chang GC. AZD9291 in EGFR inhibitor-resistant non-small-cell lung cancer. N Engl J Med. (2015) 372:1689–99. doi: 10.1056/NEJMoa1411817 25923549

[6] Li Y Peng X Lin R Wang X Liu X Meng F. Tyrosine kinase inhibitor antitumor therapy and atrial fibrillation: potential off-target effects on mitochondrial function and cardiac substrate utilization. Cardiovasc Innov Appl. (2023) 8. doi: 10.15212/cvia.2023.0070

[7] Wang X Guo X. A rare case of a primary cardiac tumor presenting as fatal ventricular tachycardia. Cardiovasc Innov Appl. (2022) 7. doi: 10.15212/cvia.2022.0014

[8] Dumani S Dibra L Likaj E Ikonomi M Kuci S Baboci A. Calcified amorphous tumor of the mitral valve causing stroke: A rare case of heart tumor. Heart Mind. (2025). doi: 10.4103/hm.HM-D-24-00053

[9] Wu YL Tsuboi M He J John T Grohe C Majem M. Osimertinib in resected EGFR-mutated non-small-cell lung cancer. N Engl J Med. (2020) 383:1711–23. doi: 10.1056/NEJMoa2027071 32955177

[10] Akamatsu H Toi Y Hayashi H Fujimoto D Tachihara M Furuya N. Efficacy of osimertinib plus bevacizumab vs osimertinib in patients with EGFR T790M-mutated non-small cell lung cancer previously treated with epidermal growth factor receptor-tyrosine kinase inhibitor: west Japan oncology group 8715L phase 2 randomized clinical trial. JAMA Oncol. (2021) 7:386–94. doi: 10.1001/jamaoncol.2020.6758 PMC7791398 33410885

[11] Zhang Y Wang X Pan Y Du B Nanthakumar K Yang P. Overdrive pacing in the acute management of osimertinib-induced ventricular arrhythmias: A case report and literature review. Front Cardiovasc Med. (2022) 9:934214. doi: 10.3389/fcvm.2022.934214 36247453 PMC9557095

[12] Anand K Ensor J Trachtenberg B Bernicker EH. Osimertinib-induced cardiotoxicity: A retrospective review of the FDA adverse events reporting system (FAERS). JACC CardioOncol. (2019) 1:172–8. doi: 10.1016/j.jaccao.2019.10.006 PMC8352117 34396179

[13] Ewer MS Tekumalla SH Walding A Atuah KN. Cardiac safety of osimertinib: A review of data. J Clin Oncol. (2021) 39:328–37. doi: 10.1200/jco.20.01171 PMC8078322 33356419

[14] Matsuura C Kato T Koyama K. Successful management of refractory torsades de pointes due to drug-induced long QT syndrome guided by point-of-care monitoring of ionized magnesium. Cureus. (2021) 13:e13939. doi: 10.7759/cureus.13939 33880279 PMC8051539

[15] Ikebe S Amiya R Minami S Ihara S Higuchi Y Komuta K. Osimertinib-induced cardiac failure with QT prolongation and torsade de pointes in a patient with advanced pulmonary adenocarcinoma. Int Cancer Conf J. (2021) 10:68–71. doi: 10.1007/s13691-020-00450-2 33489705 PMC7797397

[16] Bian S Tang X Lei W. A case of torsades de pointes induced by the third-generation EGFR-TKI, osimertinib combined with moxifloxacin. . BMC Pulm Med. (2020) 20:181. doi: 10.1186/s12890-020-01217-4 32580784 PMC7313192

[17] Kaira K Ogiwara Y Naruse I. Occurrence of ventricular fibrillation in a patient with lung cancer receiving osimertinib. J Thorac Oncol. (2020) 15:e54–5. doi: 10.1016/j.jtho.2019.11.029 32216947

[18] Jin T Hu B Chen S Wang Q Dong X Zhang Y. An *in vitro* assay of hERG K (+) channel potency for a new EGFR inhibitor FHND004. Front Pharmacol. (2018) 9:577. doi: 10.3389/fphar.2018.00577 29904349 PMC5990611

[19] Lu Z Wu CY Jiang YP Ballou LM Clausen C Cohen IS. Suppression of phosphoinositide 3-kinase signaling and alteration of multiple ion currents in drug-induced long QT syndrome. Sci Transl Med. (2012) 4:131ra150. doi: 10.1126/scitranslmed.3003623 PMC3494282 22539774

[20] Lyon AR López-Fernández T Couch LS Asteggiano R Aznar MC Bergler-Klein J. 2022 ESC Guidelines on cardio-oncology developed in collaboration with the European Hematology Association (EHA), the European Society for Therapeutic Radiology and Oncology (ESTRO) and the International Cardio-Oncology Society (IC-OS). Eur Heart J. (2022) 43:4229–361. doi: 10.1093/eurheartj/ehac244 36017568

